# Inferring sparse networks for noisy transient processes

**DOI:** 10.1038/srep21963

**Published:** 2016-02-26

**Authors:** Hoang M. Tran, Satish T.S. Bukkapatnam

**Affiliations:** 1 Department of Industrial & Systems Engineering, Texas A&M University, College Station, TX 77840, USA; 2 School of Applied Mathematics & Informatics, Hanoi University of Science & Technology, Hanoi, Vietnam

## Abstract

Inferring causal structures of real world complex networks from measured time series signals remains an open issue. The current approaches are inadequate to discern between direct versus indirect influences (i.e., the presence or absence of a directed arc connecting two nodes) in the presence of noise, sparse interactions, as well as nonlinear and transient dynamics of real world processes. We report a sparse regression (referred to as the 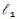-min) approach with theoretical bounds on the constraints on the allowable perturbation to recover the network structure that guarantees sparsity and robustness to noise. We also introduce averaging and perturbation procedures to further enhance prediction scores (i.e., reduce inference errors), and the numerical stability of 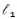-min approach. Extensive investigations have been conducted with multiple benchmark simulated genetic regulatory network and Michaelis-Menten dynamics, as well as real world data sets from DREAM5 challenge. These investigations suggest that our approach can significantly improve, oftentimes by 5 orders of magnitude over the methods reported previously for inferring the structure of dynamic networks, such as Bayesian network, network deconvolution, silencing and modular response analysis methods based on optimizing for sparsity, transients, noise and high dimensionality issues.

Many real world processes including biological^1^,^2^, socio-economics^3^,^4^, and engineering systems^5^, can be represented as large scale dynamic networks^6^. The multitude of state variables of the process represent the network nodes and the arcs represent the dynamic coupling between pairs of state variables. Inferring the structure of these networks is critical for multiple purposes such as identifying key causal relationship, clustering, partitioning or reducing the system state space; thereby facilitating effective prediction, control and/or interventions of its underlying processes. For example, inferring the signaling pathways of the gene p53 was noted to be crucial towards advancing cancer treatment^7^.

Real world processes exhibit nonlinear dynamics and they almost always occur in transient conditions. Identifying the structure, especially the existence or absence of a direct dynamic coupling between the variables of such systems has been noted to be a standing challenge of modern science^8^, and the underlying causal mechanisms remain largely undiscovered. Most often, only noisy measurements of the network outputs in the form of a small ensemble of time series data are available for network inference^8^,^9^,^10^,^11^,^12^,^13^. The use of conventional system identification approaches can produce many spurious links due to the transitivity of influences among the nodes. Several methods for network inference notably based on Bayesian update^14^,^15^,^16^,^17^,^18^,^19^, Granger causality and multivariate autoregressive^20^,^21^,^22^,^23^,^24^, partial correlation^25^, network deconvolution (ND)^26^, network silencing^27^ and conditional causal relation^28^,^29^,^30^,^31^ have been investigated to filter the effect of indirect influences. When the time series gathered under transient conditions were available, a Modular Response Analysis (MRA)^32^,^33^,^34^ method was proposed to infer the network structure at each time point. However, these methods suffer from serious drawbacks such as they mostly assume the system to exhibit linear and time-invariant dynamics^26^, determinism (noise-free)^33^,^34^,^35^, and/or the existence of a point attractor under steady state^27^. While MRA method can be employed to reconstruct dynamics under transient conditions^33^, its performance deteriorates sharply in the presence of noise and the method encounters severe numerical stability issues, especially when the underlying dynamics is highly nonlinear. This tends to severely restrict its applicability to real world processes. Notably, the earlier methods essentially focus on dealing with each of the following scenarios including transient time series^33^, noisy measurements^14^,^15^,^16^,^17^,^18^,^19^, and indirect influence removal^14^,^15^,^16^,^17^,^18^,^19^,^20^,^21^,^22^,^23^,^24^,^25^,^33^separately. The realistic scenario combining all these scenarios has not been considered. All available methods literally break down when presented with this scenario.

Towards addressing this gap, we introduce an approach based on modifying ND, silencing and MRA methods to account for sparsity, transients, noise and high dimensionality issues. Specifically, we have investigated a sparse regression (henceforth referred to as the 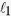-min) formulation to recover the structure of dynamic networks from noisy data gathered under transient conditions. Our main contribution is in providing a theoretical bound on the constraints of the 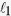-min formulation and providing stable numerical procedures that overcome effects of nonlinear couplings in large interconnected processes, availability of only a small sample of short time series ensembles, and inaccuracies in estimating noise levels. These bounds mitigate tedious trial and error procedures employed customarily as part of 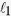-min implementations^1^,^34^,^35^,^36^. The theoretical results and subsequent experimental studies suggest that the present 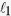-min approach is more robust to noise compared to the contemporary dynamic Bayesian network^14^,^15^,^16^,^17^,^18^,^19^ as well as NDs^26^,^27^,^32^. It is shown that up to 5 orders of magnitude reduction in the inference error are possible from the present approach, leading to a more accurate inference of the network structure for complex real world networks.

## Methods

**Figure 1:**
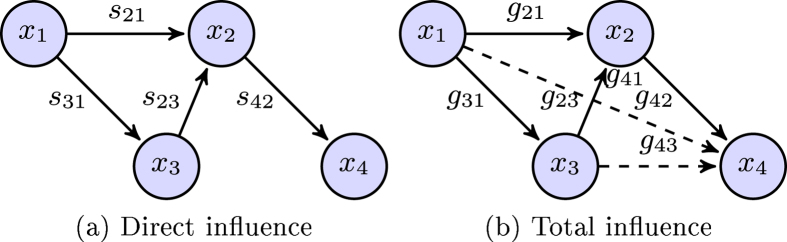
Illustration of direct and total influence. The total influences in (**b**) are the accumulation of the influences transited through all paths in (**a**). For example, the total influence 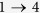 in (**b**) is the accumulation of the influence transited through the paths 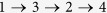 and 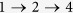.

Towards a more formal treatment, we define a real world system as high dimensional coupled differential equation of the form


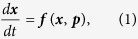


or


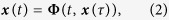


where 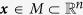 is a state vector, ***p*** is the parameter vector, 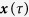 is an initial condition. As noted in the foregoing, such dynamics can also be represented in form of a network^37^ shown in Fig. 1, where the node *i* represents the state variable 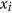 and a directed arc represents the existence and the strength of the coupling (direct influence) 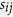 between node *i* and node *j*. In this context, the direct influence 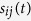 of node *j* on node *i* around a certain point ***x*** in the state space defined in Eq. (1) can be expressed as


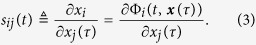


It may be noted that, a node *j* is connected to a node *i* at time *t* if 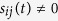. Hence, 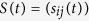 captures the physical structure of the dynamical system (1) at time *t*. In practice, 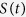 needs to be inferred from the measurements of the total influence 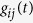 between every pair of nodes^26^,^27^ or estimated from time series outputs of the dynamic system gathered under transient conditions^33^. The total influence 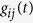 is the sum of the direct influence of node *j* on node *i* and all indirect influences from node *j* to node *i* through other nodes connecting to both of them (see Fig. 1b). For example, total influence from 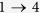, 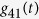 is the sum of indirect influences along the paths 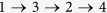 and 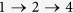, or 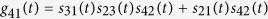. In other words, the total influence that node *j* has on node *i* around a certain point ***x*** on the state space defined in Eq. (1) is defined recursively as


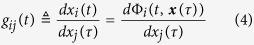



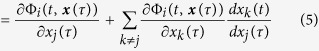



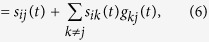


which is similar to the expression noted in in Barzel and Barabási^27^. Conventionally, under stationarity assumptions, 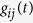 can be approximated using similarity measures, such as correlation and mutual information^8^ estimated from raw samples of time series. The direct and total influence matrices are related at every time *t* by the following equation:


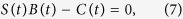


where 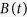 and 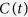 are functions (defined depending on the context) of 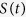 and 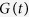, respectively. Pertinently, when the underlying dynamical system is linear and time-invariant, 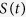 and 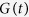 do not depend on time. Eq. (7) generalizes previous network deconvolution formulations as follows: for Feizi *et al.*^26^, 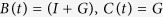, for Barzel and Barabási^27^ 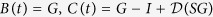 , and for Sontag *et al.*^33^, 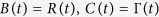, where 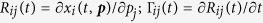. For simplicity of expressions, we use henceforth *S*, *B* and *C* instead of 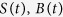 and 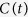 in this subsection. The “true” network structure 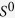 can be estimated by solving the following 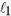-min formulation:


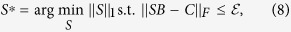


where 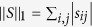, and 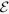 is the allowable perturbation that captures the effects of noise in the measured data. We note that in the absence of noise, this formulation is equivalent to ND and MRA. In the following sections we present two alternative 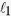-min formulations for direct influence inference. The first formulation presented in Eqs (9, 10) addresses the estimation of 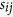 for real world scenarios when the total influence 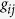 is directly measurable (e.g., based on the strengths of co-excitations), and the second formulation Eqs (21, 22) addresses the inference of the network structure (i.e., determine all node pairs where 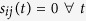) under one of the most generic scenarios of using multiple ensembles of time series realizations of the state variables, collected under noisy and transient conditions with different parameter settings. It may be noted that inferring the network structure under such generic conditions has not been investigated to date.

### Network inference when total influence matrix is available

For the case where the measurements of total influence matrix *G* are provided^26^, the relaxed 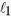-min formulation can be written as


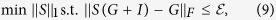


or in vector form as


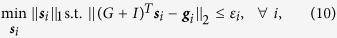


where 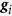 is the 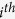 column of *G*. In order to solve for an accurate estimate of 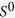 from Eqs (9) or (10) using standard solvers^38^,^39^, estimation of 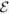 and 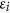 are crucial. Specifically, when noisy measurements of the total influence matrix differ from the “true” total influence as 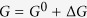, the estimated direct influence matrix differs from the true direct influence matrix as 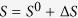, and


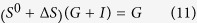



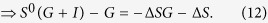


The quantity 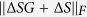 is called total perturbation. In vector form, 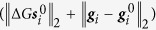 can represent the total perturbation for computing row *i* of 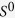. The bounds on 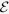 and 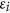 are as follows (See Theorem 1 in Supplementary Information):


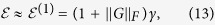



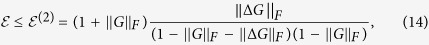



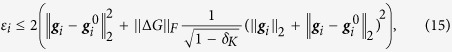


where *γ* is the largest eigenvalue of Δ*G*, *δ*_*K*_ is the restricted isometry constant^40^ and 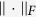 is the Frobenius norm of a matrix. By employing these bounds, we can set the values of 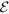 and 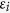 for effective network inference. As subsequent numerical investigations indicate, the performance of the method does not degrade significantly due to the presence of noise, and this is the major advantage of the present approach. It may be noted that our method is designed to provide the sparsest network structure that replicates the measured total influence *G* within a bound (specified in terms of the allowable total perturbation). This is very important because only a small set of noisy observations are available, for most real world applications. For example, in the case of genetic regulatory networks, only a subset of dynamic regimes (i.e. marked by the active degrees of freedom) of the underlying process are captured. Therefore, identification of true network structure would never be guaranteed by any approach, and among the network structures that can replicate the observed total influence within a specified bound, the sparsest network would be of the most interest. Although sparser than the network derived by ND, 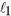-min derived structure might be adequate to uncover the total dynamic couplings of the process captured in the observed data.

In real world scenarios, 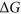 is not always known. Overestimation of 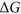 can lead to network structures that are sparser than the original. However, we show that the effects of under-estimation of noise can be alleviated to a great extent. When noise level is unknown but multiple realizations of the noisy measurements of *G* are available, it is possible to further reduce the inference error by combining the estimates with different realizations of *G* as 
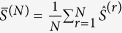
 (See the Proposition 1 in Supplementary Information), where 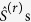 are direct influence matrices computed from 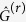 and 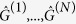 are *N* different measurements or estimates of the total influence matrix 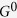. This result assumes that 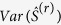 is bounded. However, it may be noted that even if 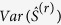 is arbitrarily large we find that 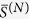 is at least as good as 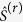. This averaging procedure allows us to improve the network inference accuracy when multiple measurements of the total influence matrix are available. For example, when the network structure does not change significantly as the system approaches a steady state, the total influence matrices can be measured multiple times, each corresponds to one time window.

### Network inference when the time series under transient conditions are available (total influence matrix not given)

In practice, 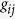 are often estimated using convenient similarity measures such as correlation or mutual information between the time series 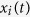 and 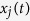 of the nodes 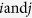 as stated in the foregoing section. These estimations have a very low accuracy due to nonstationaries (transient), low sampling rates and sample size limitation; and can not capture the total influence in the system. Also, in most real world applications, only finite samples of time series 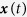 are available, and the present NDs can not be employed in these scenarios. To overcome these drawbacks, we have adapted an approach to estimate the direct influence based on multiple time series ensembles obtained by perturbing parameters of the dynamical system Eq. (1)^33^. We first modify the perturbation procedure proposed by Sontag *et al.*^33^ to make it more robust to numerical error then further improve the accuracy of network inference by introducing a sparse regression formulation and the averaging scheme.

#### A robust perturbation procedure

According to Sontag *et al.*^33^, 
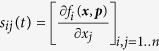
 can be derived from the following equation:


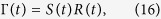


where


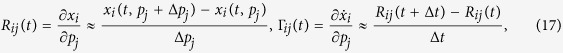


and


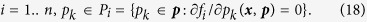


Note that Γ plays the role of the total influence matrix *G* in the previous section. To compute the row *i* of the matrix *S*, the parameters 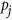 to be perturbed are chosen such that 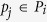
33. As a consequence, changes in 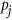 indirectly affect 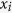, and 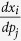 are much smaller than 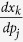, for 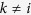. As a result, the *i*^*th*^ column 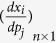 in the matrix 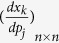 is much smaller (2 orders of magnitude smaller as in the Table 1 for the network studied in case study 1) compared to other columns when 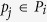. A numerical issue this poses can be understood based on the following linear system of equations


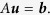


Here, the sensitivity of solution ***u*** to the change in *A* can be quantified as follows^41^


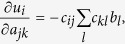


where 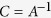. Whenever *A* contains a *j* column such that 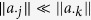, 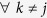, *C* contains a row *i* such that 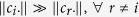. As a consequence, 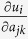 becomes several magnitudes larger than other rows. Therefore, the perturbation procedure proposed by Sontag *et al.*^33^ is very unrobust to noise or numerical error in *x*_*i*_s.

The following modification to the perturbation procedure addresses the aforementioned issue. Consider the case when 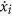 depends linearly on *x*_*i*_ as in the following system^42^:


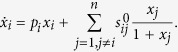


This system describes popular biochemical reactions when the activity of a chemical species is inhibited by its own concentration^43^,^44^. To compute the 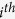 row of the Jacobian, the parameters *p*_*i*_ is also perturbed. Note that


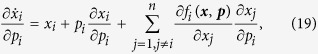


or


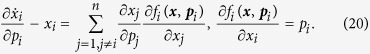


The remaining parameters are perturbed as in Eqs (17, 18). Therefore, to compute 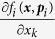, we can solve the system of equations (16) with


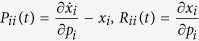


and other 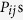, 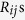 are defined as in (17, 18).

#### A robust network identification approach

In addition to the perturbation procedure proposed in Eqs (17, 18, 19), we present a method to solve Eq. (16) that is more robust to the presence of noise. In the present context, the 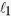-min formulation of Eq. (16) takes the following form:


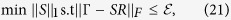


or


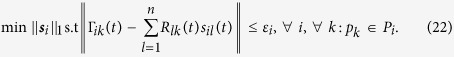


As noted in the foregoing section, estimation of 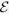 and 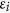 based on the noise levels when measuring 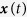 is essential to ensure that the solution to Eq. (21) serves as a viable estimator of the “true” direct influence 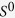. The following bounds and approximation allow the specification of 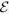 and 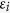 (Theorems 4 and 5 in Supplementary Information)


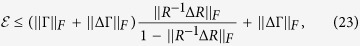



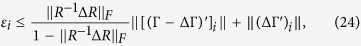



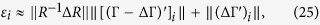


where


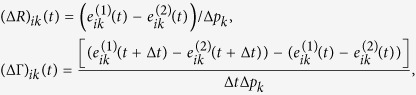


and 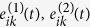 are the errors incurred when measuring 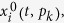 
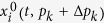
, respectively. As stated in the foregoing, noise level is not known *a priori* in most real world systems. In this situation, the network structure is deduced based on the entries in the estimated 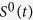 that are equal to zero for all *t* and can be estimated by the entries in as 
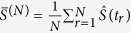
 that converge to zero, where 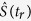 is the direct influence matrix computed from 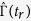, and 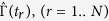 are measurements or approximations of the total influence matrix 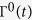 at time 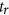 (see Proposition 2 in Supplementary Information). This averaging procedure allows us to improve the accuracy to predict the pair of nodes that are not connected when the measurement noise level is not available. As a result, our method ensures low false positive rates on the “arcs”. As noted in the context of Proposition 1, network inference with 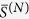 tends to be at least as good as with 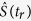 even when 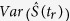 is arbitrarily large.

## Results

We have considered two case studies to validate the theoretical results and evaluate the performance of the 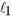-min approach. The first case study contains two simulation scenarios. The first scenario simulates a scale-free network whose structure resembles that of the genetic regulation process of *E. Coli* species^45^. Here, the challenge is to estimate the true network structure, i.e., the direct influence matrix 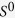 from a noisy total influence matrix *G*. This scenario is optimal for assessing the closeness of the bounds stated in Eqs (14, 15) relative to the true bounds on the constraints 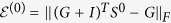, and comparing the performance of the 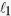-min formulation relative to the recent ND methods in terms of inference error and sparsity. The next scenario simulates a system of Hill-type differential equations modeling a gene interaction network. Here, the challenge is to estimate the true network structure from noisy and transient time series data. The second case study is an application of our method to infer genetic regulatory networks (GRNs) from empirical data in the context of DREAM5 challenge^46^. This challenge is a standard framework for evaluating GRN inference methods.

### Case I: simulation studies

#### Inferring direct influence networks from total influence network

First, we adapted the procedure specified by Muchnik^47^ to generate 500 random realizations of scale-free networks consisting of 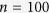 nodes, with a degree exponent of 2.2. In each realization, the weights of the true direct influence network, 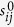 follow the distribution 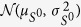 with 
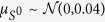
, and 
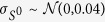
. The true total influence matrix 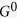 was obtained as 
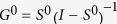
. The noisy total influence matrix was generated as 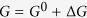, where the contaminated noise 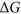 was considered in two cases: (1) proportional, i.e., 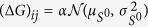 and (2) independent, i.e., 
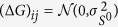
. We considered cases where the measurement noise level 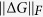 is known as well as those where there is uncertainty in estimating the measurement noise level.

We first compare the “true” bound 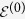 (computed using *S*^0^) and the bounds for 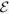 estimated based on Eqs (13, 14). In the presence of noise, the bounds appear to be in the same order of magnitude for all simulated networks (Table 2). The results also suggest that the bound specified in Eq. (13) closely matches the “true” bound and can be used to approximate the feasible region when 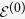 is unknown with high accuracy. Although the bound in Eq. (14) tends to be loose, it can be used as an upper bound for 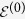.

**Figure 2:**
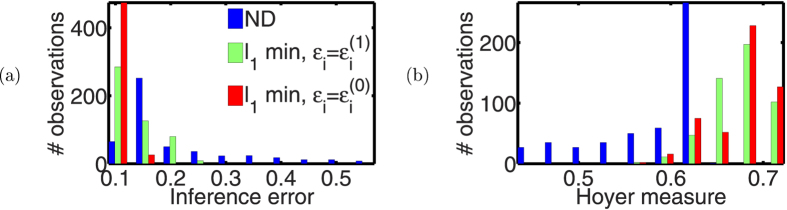
Histograms summarizing the relative performance of ND and 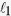-min approaches for the benchmark numerical case in terms of (**a**) inference error that quantifies the accuracy and (**b**) Hoyer measure that quantifies the sparsity of the solution. The solution from the 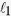-min approach is more precise and sparser than ND: compared to NDs, the mean and the variance of the inference error are reduced by 45% and 99%, respectively, when using 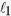-min with 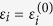; 33.5% and 87.5%, respectively when using 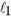-min with 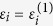; the mean of Hoyer measure is increased by 16.38% and variance reduced by 69% when using the 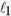-min with 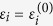, and is increased by 15.90% in mean, reduced by 75.69% in variance when using 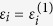.

We next compared the performance of ND and 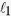-min approaches (using our bounds Eqs (13) and (14)) in terms of inference error defined as 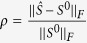, where 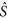 is computed using the different methods being compared. The 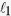-min approach with “true” constraint bound 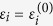 significantly improves the ND (the mean and the variance of the estimated *ρ* were reduced by 45% and 99%, respectively) (Fig. 2). Employing 
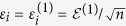
 (based on Eq. (13)), the 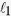-min approach performs much better than ND (the mean and variance of *ρ* are reduced by 33.5% and 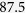
%, respectively). More importantly, the inference error of 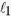-min approaches were concentrated around of 0.15 within ±0.05, while those of ND were spread over a larger range, from 0.3 to 0.6. This suggests that 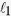-min approach using our bound in Eq. (13) is more robust than ND to noise and approximation error incurred when measuring the total influence matrix.

We also compared the sparsity of the recovered networks measured in terms of Hoyer sparsity measure^48^ defined as follows


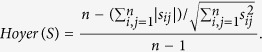


Note that 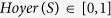. The closer it is to 1, the sparser *S* is. In terms of this measure, the solution of the 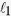-min approach is much sparser (mean is 16.38% larger, variance is 69% smaller when using the true bound 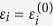, and mean is 15.90% larger, variance is 75.69% smaller when using the approximated bound 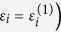 than solution of ND (Fig. 2b). Also, the Hoyer measure of the 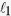-min approach is concentrated more around a much higher value (sparse matrices) than that of ND indicating that the 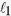-min approach using our bound gives a significantly sparser solution than ND. As a result, this gives a more interpretable connection structure without the loss of performance.

**Figure 3:**
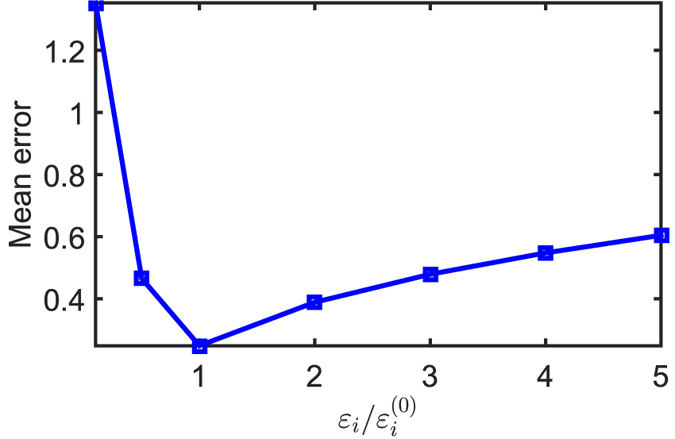
Variation of inference error with total perturbation bound *ε*_*i*_. The inference error attains a minimum near the true bound 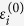, and it trends almost linearly with 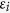 as it is increased beyond 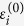. As 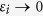, the inference error increases exponentially, which is an evidence of over fitting.

We also studied the effects of the bounds of 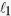-min formulation on inference error to verify Eq. (40) numerically. When 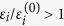, the inference error trends almost linearly with 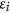 (see Fig. 3). This confirms the conclusion of Theorem 3. Also, when 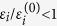 and tends toward 0, the inference error increases. This shows an evidence of over-fitting.

**Figure 4:**
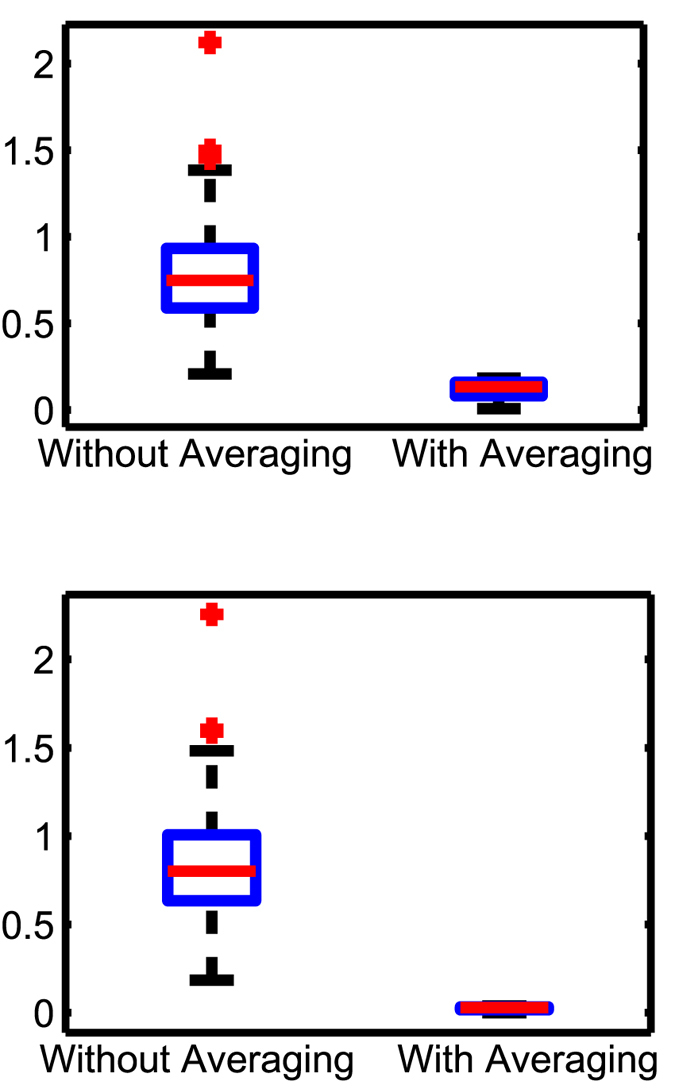
Box plots summarizing the effects of averaging on (**a**) ND and (**b**) 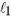-min with 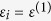. The inference errors were almost unchanged with 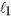-min compared to ND. Averaging (light/red) reduced inference error further by about 8 times compared to without averaging (dark/blue). The 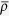 values were 0.1196 with ND and 0.0259 with 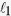-min (p-values of the paired t-tests between the inference error without and with averaging were ≤10^−5^ in all cases).

Subsequently, we studied the effect of averaging (Proposition 1) in the context of the 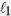-min and ND methods. We conducted *N* = 40 simulations, in each of which, 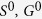 and 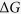 were generated as stated in the foregoing. We used the inference error without 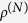 and with averaging 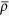 as measures for comparison from each simulation defined as follows:


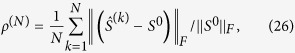



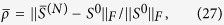


where Ŝ^(k)^ 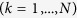 is the 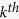 realization of 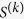 and 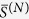 is estimated as stated in Proposition 1. The results suggest that averaging reduces the inference error of both methods by about 8 times in all cases, thus supporting the validity of Proposition 1 (Fig. 4). The inference errors were almost the same between ND and 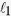-min with 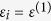.

### Inferring direct influence network structure from multiple time series under transient conditions

In this section we represent the performance of 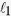-min approach in inferring network structure from transient time series with an unknown noise level. In this study we used Michaelis-Menten dynamic system given by^27^:


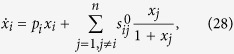


where the “true” network defined by 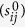 is a scale-free network^45^ generated randomly with degree exponent 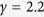 consisting of 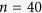 nodes with about 70 edges, whose weights 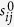 follow the distribution 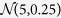.

We obtained 30 different variants of this network. For each of these invariants (trials), a perturbed network was obtained by changing (perturbing) the parameters according to Eqs (18, 19, 20). Every solution 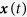, 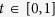, obtained from an initial condition 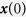 was contaminated with noise of the form 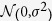 to simulate a noisy measurement 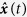. Here 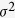 was chosen to be 10^−4^. The direct influence matrix 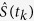 were estimated using Sontag *et al.*’s^33^ method, as well as 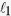-min formulations, with different values of bounds. Next, 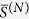 was estimated as in Proposition 2 by averaging over 30 time samples 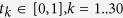 chosen randomly. For performance evaluation, we used the inference error without 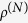 and with averaging 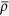, given by


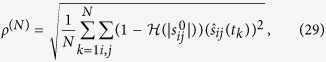



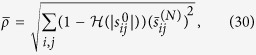


where 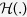 is Heaviside function. These error measures quantify the number of absent links 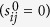 that are correctly identified.

**Figure 5:**
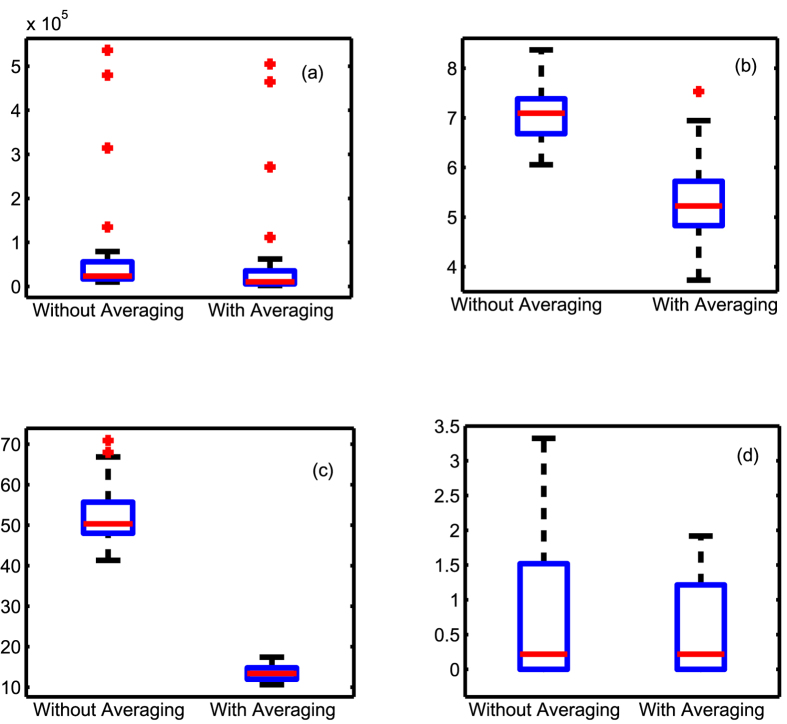
Box plots summarizing the inference errors without and with averaging for. (**a**) Sontag *et al.*’s^33^ method 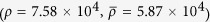; (**b**) 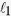-min with noise magnitude given 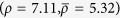, (**c**) 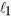-min with noise magnitude underestimated as 10% the actual 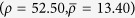, and (**d**) 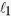-min with noise magnitude overestimated as 10 times the actual 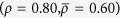. The inference error was reduced by 10^5^ times when using the 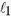-min approach (21, 22), compared to Sontag *et al.*’s^33^ method. Averaging further reduced inference error by at least 30% in all cases (p-values of the paired t-tests consistently were below 0.0282).

As summarized in Fig. 5, the 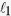-min approach performs better than Sontag *et al.*’s^33^ method in all cases tested. In fact, 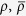 were reduced by 10^5^ times. The poor performance of Sontag *et al.*’s^33^ method is attributed to the numerical issues noted in the earlier section. A further 30% reduction in inference error resulted from averaging for both cases. Next, the cases (c) and (d) were designed to simulate the real situations where the noise magnitude is unknown. We considered cases where the noise levels are under or overestimated by 1 order of magnitude. While Sontag *et al.*’s^33^ method would not be applicable in such cases, 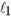-min without averaging was found to lead to suboptimal inference. Under underestimation 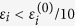, averaging was found to further reduce the inference error by about 70%, and the inference error 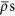 were of the same level as one would obtain when the noise level is known. This result is consistent with and is a clear verification of Proposition 2. When the noise level is overestimated, the resulting network tends to be highly sparse, offering excellent specificity in identifying the absence of direct coupling. The inference errors are therefore low even without averaging by default. In this case averaging reduces the inference errors by 5%. The p-values of the paired t-tests between the inference error with and without averaging were below 0.0282 in all cases suggesting that averaging helps improve network inference.

#### Case II: Application to empirical genetic regulatory network inference

Next, we applied our method to infer real world GRNs and compare its performance with other methods including ND^26^, Bayesian network inference, Pearson and Spearman correlation networks^8^ using the framework presented in DREAM5 challenge. Here, the Pearson and Spearman correlations were considered as they are the most widely used methods for network inference and can provide a reasonable estimation of the total influence matrix^26^,^27^. In addition, ND has been most effective in inferring network topology when the total influence matrix G is estimated using Person and Spearman correlations. Therefore, these serve as the challenging test cases to evaluate the performance of 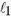-min where ND is already effective. The DREAM5 challenge contains gene-expression microarray data of three species including an *in silico* benchmark, a prokaryotic model organism (*E. coli*) and a eukaryotic model organism (*S. cerevisiae*). Beside *ρ* and Hoyer metrics, we employed the following score, which was used in earlier works^8^ to assess the performance of a network inference method for recovering the structure underlying these data sets:


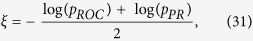


where 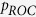 and 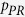 are p-values computed from AUROC (area under receiver operating characteristic curve) and AUPR (area under precision-recall curve).

**Figure 6:**
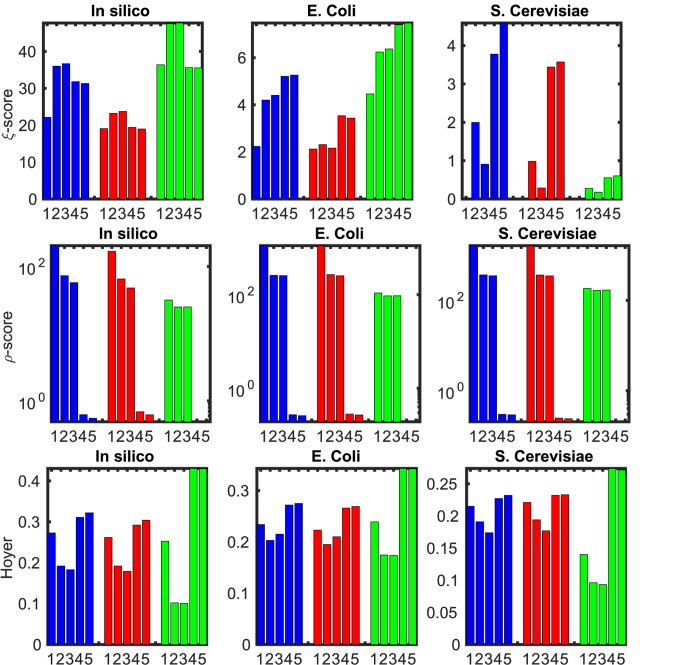
Performance comparison of (1) original *G* matrix, (2) ND, (3) ND with averaging, (4) *ℓ*_1_-min and (5) *ℓ*_1_-min with averaging for the DREAM5 challenge datasets. The total influence *G* matrix is estimated by Pearson correlation (blue/dark), Spearman correlation (red/light) and Mutual Information (green/light). Compared to ND, the prediction scores with 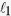-min are increased by 23.94% (for *G* from Pearson correlation), 53.03% (for *G* from Spearman correlation) & 18.53% (for *G* from Mutual Information) for *E. Coli*, 89.09%, 249.7% & 116.74% for *S. cerevisiae*, respectively; the inference errors *ρ* (29) are reduced by 2 to 3 orders of magnitude in all cases; Hoyer measures are increased by 34%, 36.41% & 322.91% for *E. Coli*, 18.85%, 19.59% & 96.65% for *S. cerevisiae*, respectively. For *in silico* data, ND gives a solution with 11% higher prediction score but 33% less sparse than 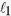-min approach. Averaging slightly improves the performance of all methods (<10%).

The results of the performance evaluation are summarized in Fig. 6. We note that for computing the performance metrics we first generated 30 different *G* matrices with Pearson correlation, 30 others with Spearman correlation and another 30 with Mutual Information for each data set. The *G* matrix in each case was estimated using samples of size 75% of the data set. The averaging procedure considers the *S* matrices estimated from these *G* matrices using different methods. In terms of *ξ*-score (Eq. (31)), which quantifies how well—in terms of having low false negative rates (FNR, related to sensitivity), and low false positive rates (FRN, related to specificity), the true positive rate (TPR) and true negative rate (TNR)—the estimated 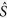 captures 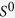, 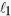-min approach yields 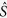 with at least 18.53% higher than with ND in all cases tested except the *in silico* case (see Fig. 6). Both ND and 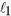-min performed better than Bayesian network approach whose *ξ*-scores were 14.891, 0.029, 0.0001, respectively, for the three data sets^8^. In terms of *ρ*-score (Eq. (29)), which quantifies the false positive rates (i. e., the specificity), 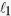-min approach reduces *ρ* by 2-3 orders compared to ND in all cases. These results provide a strong evidence for the relevance of the 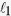-min approach for network structure inference. In terms of sparsity, 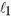-min approach increased the Hoyer measure by about 20% in most cases, and were much closer to the Hoyer measures of the gold-standard network, compared to ND.

As noted earlier for *in silico* data, although the *ρ*-score with 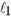-min was at least 1160% lower (i.e., higher specificity) and Hoyer was 33% higher (i.e., higher sparsity), the *ξ*-score was slightly (10%) lower than with ND. The lower *ξ*- score for 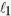-min is perhaps a consequence of the method being susceptible to over-specification of the noise level. In this context, it must be noted that the solutions from both ND and 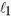-min can replicate the observed total influence *G* within a specified bound (as total perturbation). However, the solutions from 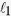-min tend to be much sparser and have lower false positive rate. Given that there were only 805 sample measurements to reconstruct *G* matrices for 1643 nodes in the *in silico* network, it is highly likely that several dynamic modes (degree of freedom) are not observable from the data. Therefore, 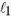-min generated a much sparser network which, by formulation, is guaranteed to be adequate to capture the observed modes of the dynamics within the specified total perturbation limits. The ND derived networks for *in silico* and other cases that have higher *ξ*-score, intriguingly, were consistently found to have much lower Hoyer score (hence sparsity) even compared to the specified total influence matrix. Thus, 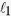-min-generated solutions provide significant improvement in specificity, although the sensitivity at times were found to be slightly lower than with ND.

Averaging improves the *ξ*-scores (Eq. (31)) with all methods by at most 10%. This is perhaps due to the near-stationarity of the total influence matrix *G*, when computed using data over long time windows that smooths out various higher order transient effects. Also, one may note that the averaging makes the network inferred from ND less sparse than without averaging. This is because under noise, transients and data sparsity, ND yields vastly different network topologies depending on the samples employed. Averaging over these vastly different networks causes a reduction in sparsity. These results, taken together suggest that the 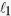-min approach is perhaps the best known means to provide specificity for network inference from transient and noisy data. The utility of the approach would be to provide a minimal set of arcs (dynamic couplings or direct influences) to be considered for further network dynamics reconstruction applications.

### Discussion and Concluding remarks

In this paper, we have investigated a method to robustly infer the structure of a network representing a sparse dynamical system from noisy, transient time series data. When the noise level is known, the 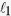-min formulation employing our theoretical formula for the bound on total perturbation improves the recently reported NDs in terms of both accuracy and sparsity. When the noise level is unknown, we have shown that by averaging the networks inferred from different time points or conditions, the inference of network structure of real world processes becomes highly plausible.

Pertinently, for most real world processes, the total influence is not known a priori; only the time series ensembles gathered under transient conditions are available (e.g., gene expression microarray data^8^,^49^, protein-protein interaction data^50^ as in the case of Michaelis-Menten dynamics). It has been noted that most of the earlier approaches present severe accuracy, noise sensitivity and/or numerically stability issues for such realistic scenarios. To overcome these limitations, we have investigated the 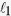-min approach with a novel perturbation procedure for time series based network inference. Averaging over the solutions estimated at different time windows has been shown to allow inference of the structure for complex real world networks, especially when the noise levels are unknown or cannot be accurately estimated.

Next, we have applied our method to three benchmark systems: a sparse scale-free network^51^ with a specified noise level and the total influence between any two nodes given, a genetic regulatory network model formulated in terms of a system of Hill-type differential equations^27^, and GRNs of DREAM5 challenge^46^. These analyses suggest that our proposed bounds on the constraints for the 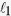-min formulation, extracted from a few time series samples acquired under transient conditions, are of the same order (i.e., they closely envelop) with the constraints estimated based on the full knowledge of the noise level. The 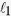-min formulation reduces the inference errors defined in (31) and (29) by 18.53% and 2 to 3 orders of magnitude, respectively, and improves the sparsity of the solution (measured in terms of Hoyer sparsity measure) by 15.9%, in comparison with conventional approaches including various versions of dynamic Bayesian approaches for network inference as well as ND. If instead of the total influence, only the time series gathered under transient conditions is provided, such as in the case of Michaelis-Menten dynamics, 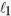-min approach achieves a 4 order reduction in inference error compared to MRA. These theoretical and and numerical studies suggest that our proposed method can be employed to effectively infer the presence of dynamic coupling (i.e., arc set or the direct influence in a dynamic network) based on sparse samples.

As with any network reconstruction approach, the method assumes that the time series realizations taken together can adequately mirror the salient dynamic regimes of the underlying process^52^, and as noted earlier, the approach is restricted to ensuring high levels of specificity and not sensitivity in identifying the direct influences. Additionally, while the approach is fairly robust to the presence of noise, the estimates 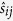 from the averaging procedure for the arcs with 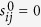 is guaranteed to converge to zero only in the presence of additive noise. More specifically, one of the following conditions need to hold for the approach to be applicable: (1) the governing equation of the process dynamics is specified, so that 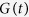 or 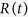 can be constructed; (2) one or more realizations of 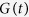 (based on ND or silencing method) or 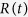 (based on MRA) are given. In our experience, 30 realizations ensured the convergence of the averaging method; (3) one realization of a *n*-dimensional time series is available for estimating 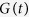 using various alternative methods outlined in Feizi *et al.*’s^26^ or 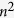 time series realizations with the same initial condition are available for estimating 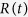 using Eq. (17). Note that Scenario 1 is useful only for applications such as to investigate if there exists a more compact (sparser) network representation to capture the specified process dynamics. In Scenarios 2 and 3, we assume that the noise level or its lower limit is known, and adequate number of realizations are available to ensure convergence of the averaging method. In scenario 3, Eq. (17) yields a finite space-time approximation of the partial derivatives 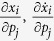. They are estimated by perturbing the parameters 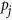 and keeping the initial condition the same for two time series signals. The length of the time series in this case can be really small, or it can just be samples taken over multiple (roughly 30), short (can be even 2 samples) time windows. However, the time steps (or sampling interval) in each time window must be small enough to ensure that 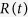 values locally converge. Sensitivity of the network inference performance to time step size, however, needs further investigation.

Efforts are underway to address some of the 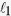-min aforementioned limitations. We are investigating a two-stage approach to recover local nonlinear dynamics from sparse time series data. For future research, we will consider a more realistic scenario where not all state variables can be measured. In GRN inference, for example, only the outputs/activations of only those genes that have been discovered are measured. However, unknown genes might have significant influence on the network structure. Removing the effects of unmeasured variables, when combined with the method proposed in this paper, will lead to a more advanced network inference method.

## Additional Information

**How to cite this article**: Tran, H. M. and Bukkapatnam, S. T.S. Inferring sparse networks for noisy transient processes. *Sci. Rep.* **6**, 21963; doi: 10.1038/srep21963 (2016).

## Supplementary Material

Supplementary Information

Supplementary Information

## Figures and Tables

**Table 1:** The matrix *R* for computing the first row of *S* is estimated using Sontag *et al.* 33’s perturbation procedure.

	*r*._1_	*r*._2_	*r*._2_	*r*._2_	*r*._2_	*r*._2_	*r*._2_	max|*r*._*j*_|
*r*_1._	−2.868e-4	−7.284e-5	−3.106e-4	−1.578e-4	2.443-e4	−8.315e-5	−4.896e-4	**0.0005**
*r*_2._	1.160e-4	0.1050	−4.261e-4	−1.803e-4	6.490e-4	2.261e-4	−2.379e-4	***0.1050***
*r*_3._	−1.136e-4	−2.658e-4	0.1179	−2.766e-4	1.370e-4	−2.524e-4	2.776e-4	***0.1179***
*r*_4._	−4.431e-4	−4.543e-4	−4.138e-4	0.0961	6.824e-4	6.710e-5	1.609e-4	***0.0961***
*r*_5._	−2.397e-4	−4.439e-4	−1.225e-4	−7.024e-4	0.1100	3.256e-4	2.069e-4	***0.1100***
*r*_6._	4.053e-4	−3.773e-4	−2.577e-4	−5.065e-5	0.0012	0.1195	4.481e-4	***0.1195***
*r*_7._	−1.030e-4	3.312e-5	−2.900e-4	−5.258e-5	0.0100	−1.651e-4	0.0820	***0.0820***

The first row/column of *R* is two orders of magnitude smaller than others, which presents major numerical issues for inferring structures of large networks.

**Table 2:** Comparison of bounds on total perturbation obtained using Eqs (13) and (14) suggests that Eq. (13) provides a good approximation and Eq. (14) serves as an upper bound of 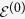.

Formula	Mean
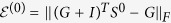	9.79 × 10^−3^
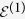 (Eq. 13)	8.89 × 10^−3^
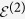 (Eq. 14)	8.61 × 10^−2^
