# Supplementary material for: Inferring sparse networks for noisy transient processes
Source: Sci Rep. 2016 Feb 26;6:21963. doi: 10.1038/srep21963 (PMC4768174; doi:10.1038/srep21963)
Supplement: Supplementary Information [file srep21963-s1.pdf]

Supplementary information:

Inferring sparse networks for noisy transient processes

Hoang M. Tran[1,2], Satish T.S Bukkapatnam[1]

1 Department of Industrial & Systems Engineering, Texas A&M University, College Station, TX 77840, USA

2 School of Applied Mathematics & Informatics, Hanoi University of Science & Technology, Hanoi, Vietnam

Theoretical results in the Section "network inference when total influence matrix is available"

Lemma 1. *$||\Delta S||_F$ can be bounded as*

$$||\Delta S||_F \leq \gamma + O(\delta^2 + \gamma^2 + \delta\gamma), \tag{32}$$

according to Feizi et al.[26] where γ and δ are the largest eigenvalues of ΔG and G, respectively satisfy $\gamma \ll 1, \delta < 1$ or

$$||\Delta S||_F \leq \frac{||\Delta G||_F}{(1-||G||_F-||\Delta G||_F)(1-||G||_F)}, \tag{33}$$

provided

$$1-||G||_F-||\Delta G||_F > 0. \tag{34}$$

Proof. From (11), it follows that

$$\begin{aligned}
G^0+\Delta G &= S^0+S^0G^0+S^0\Delta G \\
&\quad +\Delta S+\Delta S G^0+\Delta S\Delta G \\
\Leftrightarrow \Delta G &= S^0\Delta G+\Delta S+\Delta S G^0+\Delta S\Delta G \\
\Leftrightarrow \Delta G &= G^0(I+G^0)^{-1}\Delta G+\Delta S(I+G^0+\Delta G) \\
\Rightarrow \Delta S &= ((I+G^0)(I+G^0)^{-1}-G^0(I+G^0)^{-1}) \\
&\quad \Delta G(I+G^0+\Delta G)^{-1} \\
\Rightarrow \Delta S &= (I+G^0)^{-1}\Delta G(I+G^0+\Delta G)^{-1} \\
\Rightarrow ||\Delta S||_F &= ||(I+G^0)^{-1}\Delta G(I+G)^{-1}||_F
\end{aligned}$$

$$
\begin{aligned}
&= ||(I-(-G^0))^{-1}\Delta G(I-(-G))^{-1}||_F \\
&\overset{a}{\leq} ||(I-(-G^0))^{-1}||_F||\Delta G||_F||(I-(-G))^{-1}||_F \\
&\overset{b}{\leq} \frac{1}{1-||(-G)||_F}||\Delta G||_F\frac{1}{1-||G||_F} \\
&\overset{c}{\leq} \frac{1}{1-||G||_F-||\Delta G||_F}||\Delta G||_F\frac{1}{1-||G||_F}
\end{aligned}
$$

We have (a) because of the sub-multiplicative property $||AB||_F \leq ||A||_F||B||_F$. for (b) to hold:

$$
||G^0||_F < 1 \tag{35}
$$

$$
||-G||_F < 1 \tag{36}
$$

Because $||G^0||_F = ||G-\Delta G||_F \leq ||G||_F + ||\Delta G||_F$, sufficient condition for (35,36) to hold is $||G||_F + ||\Delta G||_F \leq 1$.

We have (c) because

$$
\begin{aligned}
1-||G^0||_F &\geq 1-||G||_F-||\Delta G||_F > 0 \\
\Rightarrow \frac{1}{1-||G^0||_F} &\leq \frac{1}{1-||G||_F-||\Delta G||_F}
\end{aligned}
$$

Therefore, we have (33). □

Note that the restriction (34) is reasonable as G can be linearly scaled[26] such that $||G||_F$ is small enough to qualify Eq. (34). The following theorem provides bounds on total perturbation based on this lemma.

Theorem 1. *ε_i and $\mathscr{E}$ can be bounded as follows*

$$
\varepsilon_i = (||\Delta G\boldsymbol{s}_i^0||_2 + ||\boldsymbol{g}_i - \boldsymbol{g}_i^0||_2)^2 \leq 2(||\boldsymbol{g}_i - \boldsymbol{g}_i^0||_2^2 + ||\Delta G||_F\frac{1}{\sqrt{1-\delta_K}}(||\boldsymbol{g}_i||_2 + ||\boldsymbol{g}_i - \boldsymbol{g}_i^0||_2)^2), \tag{15}
$$

$$
\mathscr{E} = ||\Delta SG + \Delta S||_F \approx \mathscr{E}^{(1)} = (1+||G||_F)\gamma \tag{13}
$$

and

$$
\mathscr{E} = ||\Delta SG + \Delta S||_F \leq \mathscr{E}^{(2)} \tag{14}
$$

where $\mathscr{E}^{(2)} = (1+||G||_F)\frac{||\Delta G||_F}{(1-||G||_F-||\Delta G||_F)(1-||G||_F)}$.

Proof. Apply the Lemma 2 of Herman & Strohmer[35] to $\Phi^0 = G^0 + I$ and K - sparse vector $\boldsymbol{s}_i^0$, we have

$$
||(G^0+I)\boldsymbol{s}_i^0||_2 \geq \sqrt{1-\delta_K}||\boldsymbol{s}_i^0||_2.
$$

Also, by applying the Cauchy Schwarz inequality, we have $||\Delta G\boldsymbol{s}_i^0||_2 \le ||\Delta G||_F||\boldsymbol{s}_i^0||_2$. Therefore,

$$\begin{aligned}
\frac{||\Delta G\boldsymbol{s}_i^0||_2}{||(G^0+I)\boldsymbol{s}_i^0||_2} &\le \frac{||\Delta G||_F||\boldsymbol{s}_i^0||_2}{\sqrt{1-\delta_K}||\boldsymbol{s}_i^0||_2} \\
\Rightarrow \frac{||\Delta G\boldsymbol{s}_i^0||_2}{||(G^0+I)\boldsymbol{s}_i^0||_2} &\le \frac{||\Delta G||_F}{\sqrt{1-\delta_K}} \\
\Rightarrow ||\Delta G\boldsymbol{s}_i^0||_2 &\le \frac{||\Delta G||_F}{\sqrt{1-\delta_K}}||\boldsymbol{g}_i^0||_2
\end{aligned}$$

As a result,

$$\begin{aligned}
2(||\Delta G\boldsymbol{s}_i^0||_2^2+||\boldsymbol{g}_i-\boldsymbol{g}_i^0||_2^2) &\le 2\left(\left(\frac{||\Delta G||_F}{\sqrt{1-\delta_K}}||\boldsymbol{g}_i^0||_2\right)^2+||\boldsymbol{g}_i-\boldsymbol{g}_i^0||_2^2\right) \\
&\le 2\left(\left(\frac{||\Delta G||_F}{\sqrt{1-\delta_K}}(||\boldsymbol{g}_i||_2+||\delta\boldsymbol{g}_i||_2\right)^2+||\boldsymbol{g}_i-\boldsymbol{g}_i^0||_2^2\right)
\end{aligned}$$

On the other hand,

$$2(||\Delta G\boldsymbol{s}_i^0||_2^2+||\boldsymbol{g}_i-\boldsymbol{g}_i^0||_2^2) \ge (||\Delta G\boldsymbol{s}_i^0||_2+||\boldsymbol{g}_i-\boldsymbol{g}_i^0||_2)^2$$

Therefore, we have (15).

Proof of (13):

$$\begin{aligned}
||\Delta SG+\Delta S||_F &\le ||\Delta SG||_F+||\Delta S||_F \\
&\le ||\Delta S||_F||G||_F+||\Delta S||_F \\
&\approx \gamma(1+||G||_F)
\end{aligned}$$

as

$$||\Delta S||_F \approx \gamma, \tag{37}$$

according to.[27]

Proof of (14):

$$\begin{aligned}
||\Delta SG+\Delta S||_F &\le ||\Delta S||_F||G||_F+||\Delta S||_F \\
&\le (1+||G||_F)\frac{||\Delta G||_F}{(1-||G||_F-||\Delta G||_F)(1-||G||_F)}
\end{aligned}$$

□

Next we show that S^* obtained based on the foregoing results is a good approximation of S^0.

Theorem 2. [35] *Assume that $\boldsymbol{s}_i^0$ is the sparsest solution of the Problem (10) and*

$$\delta_{2K} < \frac{\sqrt{2}}{(1+\varepsilon_{\Phi^0}^{(2K)})^2} - 1,$$

there exists positive constants C_0, C_1 such that

$$||\boldsymbol{s}_i^* - \boldsymbol{s}_i^0||_2 \leq \frac{C_0}{\sqrt{K}}||\boldsymbol{s}_i^0 - \boldsymbol{s}_i^{(K)}||_2 + C_1\varepsilon_i \tag{38}$$

where $\boldsymbol{s}_i^*$ is solution of the ℓ_1-min problem (10).

Proof. This is a direct application of Theorem 2 in Herman & Strohmer[35] with $\boldsymbol{x} = \boldsymbol{s}_i^0, \hat{\boldsymbol{b}} = \boldsymbol{g}_i, \boldsymbol{z}^* = \boldsymbol{s}_i^*, \hat{A} = G + I$. Note that C_0, C_1 are constants depending on $\varepsilon_{\Phi^0}^{(2K)}$. □

When the true solution $\boldsymbol{s}_i^0$ has at most K nonzero elements, the result Eq. (38) can be further simplified as follows.

Corollary 1. *When $\boldsymbol{s}_i^0$ has at most K nonzero elements,*

$$||\boldsymbol{s}_i^* - \boldsymbol{s}_i^0||_2 \leq C_1\varepsilon_i. \tag{39}$$

Proof. When $\boldsymbol{s}_i^0$ is a K - sparse vector, $\boldsymbol{s}_i^0 = \boldsymbol{s}_i^{(K)}$. Eq. (38) becomes Eq. (39). □

The assumption in this corollary is reasonable since most of real world networks tend to be sparse. The results in Eqs. (38,39) are formulated for each row of S^0. In terms of the whole matrix, the robustness of computing S^0 can be guaranteed by the following theorem.

Theorem 3. *Let S^* be the solution of the ℓ_1-min formulation (9). The error when approximating S^0 by S^* is bounded by*

$$||S^* - S^0||_F^2 \leq C_1\mathscr{E} \tag{40}$$

where $\mathscr{E}$ is bounded as $\mathscr{E} \leq 2\left(\frac{1}{1-\delta_K}||G^0||_F^2 + 1\right)||\Delta G||_F^2$.

Proof. Apply the Corollary 1 to the Problem 10 with $\varepsilon_i = 2\left(||\Delta G||_F \frac{1}{\sqrt{1-\delta_K}}||\boldsymbol{g}_i^0||_2\right)^2 + 2||\boldsymbol{g}_i - \boldsymbol{g}_i^0||_2^2$ we have

$$\begin{aligned}
||\boldsymbol{s}_i^* - \boldsymbol{s}_i^0||_2^2 &\leq 2C_1\left(||\Delta G||_F \frac{1}{\sqrt{1-\delta_K}}||\boldsymbol{g}_i^0||_2\right)^2 + 2C_1||\boldsymbol{g}_i - \boldsymbol{g}_i^0||_2^2 \\
\Rightarrow \sum_{i=1}^n ||\boldsymbol{s}_i^* - \boldsymbol{s}_i^0||_2^2 &\leq 2C_1\sum_{i=1}^n\left(||\Delta G||_F \frac{1}{\sqrt{1-\delta_K}}||\boldsymbol{g}_i^0||_2\right)^2 + 2C_1\sum_{i=1}^n||\boldsymbol{g}_i - \boldsymbol{g}_i^0||_2^2 \\
&= 2C_1\left(||\Delta G||_F \frac{1}{\sqrt{1-\delta_K}}\right)^2\sum_{i=1}^n\left(||\boldsymbol{g}_i^0||_2\right)^2 + 2C_1\sum_{i=1}^n||\boldsymbol{g}_i - \boldsymbol{g}_i^0||_2^2 \\
\Rightarrow ||S^* - S^0||_F^2 &\leq 2C_1\left(||\Delta G||_F \frac{1}{\sqrt{1-\delta_K}}\right)^2||G^0||_F^2 + 2C_1||\Delta G||_F^2.
\end{aligned}$$

□

Theorems 2, 3 and Corollary 1 taken together guarantee that inference error when estimating S^0 by S^* is at most linear with total perturbation noise. This observation is further verified using numerical investigations presented in the first case study.

Proposition 1. *Let $\hat{G}^{(1)},...,\hat{G}^{(N)}$ be N different measurements or estimates of the total influence matrix G^0. Let $\hat{S}^{(r)}$ be the direct influence matrix computed from $\hat{G}^{(r)}$ using different methods, including ND and ℓ_1-min approach with different bounds. If $Var(\hat{S}^{(r)})$ are bounded then $E||\bar{S}^{(N)} - S^0||^2 \to 0$ as $N \to \infty$, where $\bar{S}^{(N)} = \frac{1}{N}\sum_{r=1}^{N}\hat{S}^{(r)}$.*

Proof. Under perfect reconstruction per ND, $\hat{S} = S^0 + \Delta S^*$ satisfies

$$\hat{S}(G^0 + \Delta G + I) = (G^0 + \Delta G)$$

For the rth realization of ΔG, we have

$$\begin{aligned} \hat{S}_r(G^0 + \Delta G_r + I) &= (G^0 + \Delta G_r) \\ \Rightarrow \hat{S}_r &= (G^0 + \Delta G_r)(G^0 + \Delta G_r + I)^{-1} \end{aligned}$$

As ΔG_r are independent, $\hat{S}_r$ are independent.

$$\begin{aligned} Var(\bar{S}^{(N)} - S^0) &= Var(\frac{\sum_{r=1}^{N}\hat{S}_r}{N} - S^0) \\ &= Var(\frac{\sum_{r=1}^{N}(\hat{S}_r - S^0)}{N}) \\ &= \frac{1}{N^2}Var(\sum_{r=1}^{N}(\hat{S}_r - S^0)) \\ &= \frac{1}{N^2}\sum_{r=1}^{N}Var(\hat{S}_r - S^0) \text{ (As } \hat{S}_r - S^0 \text{ are independent)} \end{aligned}$$

If $Var(\hat{S}_r - S^0)$ is bounded by some constant C for all r,

$$Var(\bar{S}^{(N)} - S^0) \le \frac{1}{N^2}\sum_{r=1}^{N}C = \frac{C}{N} \to 0 \text{ as } N \to \infty$$

□

Theoretical results in the Section "Network inference when the time series under transient conditions are available (total influence matrix not given)"

The errors in the estimation of R, Γ can be expressed based on the following lemma

Lemma 2.

$$(\Delta R)_{ik}(t) = (e^{(1)}_{ik}(t) - e^{(2)}_{ik}(t))/\Delta p_k$$
$$(\Delta \Gamma)_{ik}(t) = \frac{[(e^{(1)}_{ik}(t+\Delta t) - e^{(2)}_{ik}(t+\Delta t)) - (e^{(1)}_{ik}(t) - e^{(2)}_{ik}(t))]}{\Delta t \Delta p_k}$$

where $e^{(1)}_{ik}(t), e^{(2)}_{ik}(t)$ are the errors incurred when measuring $x^0_i(t, p_k)$, $x^0_i(t, p_k + \Delta p_k)$, respectively.

Proof.

$$\begin{aligned}
R^0_{ik}(t) &\approx (x^0_i(t, p_k + \Delta p_k) - x^0_i(t, p_k))/\Delta p_k \qquad (41)\\
R_{ik}(t) &\approx ((x^0_i(t, p_k + \Delta p_k) + e^{(2)}_{ik}(t)) - (x^0_i(t, p_k) + e^{(1)}_{ik}(t)))/\Delta p_k\\
&= ((x^0_i(t, p_k + \Delta p_k) - x^0_i(t, p_k)) + (e^{(2)}_{ik}(t) - e^{(1)}_{ik}(t)))/\Delta p_k\\
&\approx R^0_{ik}(t) + (e^{(2)}_{ik}(t) - e^{(1)}_{ik}(t))/\Delta p_k\\
\Delta R_{ik}(t) &= (e^{(2)}_{ik}(t) - e^{(1)}_{ik}(t))/\Delta p_k
\end{aligned}$$

$$\begin{aligned}
\Gamma_{ik}(t) &\approx (R_{ik}(t+\Delta t) - R_{ik}(t))/\Delta t\\
&= \left[R^0_{ik}(t+\Delta t) + (e^{(2)}_{ik}(t+\Delta t) - e^{(1)}_{ik}(t+\Delta t))/\Delta p_k\right]/\Delta t -\\
&\quad \left[R^0_{ik}(t) + (e^{(2)}_{ik}(t) - e^{(1)}_{ik}(t))/\Delta p_k\right]/\Delta t\\
&= \left[R^0_{ik}(t+\Delta t) - R^0_{ik}(t)\right]/\Delta t +\\
&\quad \left[(e^{(2)}_{ik}(t+\Delta t) - e^{(1)}_{ik}(t+\Delta t)) - (e^{(2)}_{ik}(t) - e^{(1)}_{ik}(t))\right]/(\Delta t \Delta p_k)\\
&= \Gamma^0_{ik}(t) + \left[(e^{(2)}_{ik}(t+\Delta t) - e^{(1)}_{ik}(t+\Delta t)) - (e^{(2)}_{ik}(t) - e^{(1)}_{ik}(t))\right]/(\Delta t \Delta p_k)\\
\Delta \Gamma_{ik}(t) &= \left[(e^{(2)}_{ik}(t+\Delta t) - e^{(1)}_{ik}(t+\Delta t)) - (e^{(2)}_{ik}(t) - e^{(1)}_{ik}(t))\right]/(\Delta t \Delta p_k)
\end{aligned}$$

□

Based on this lemma, the total perturbation can be estimated by the following theorem.

Theorem 4. *The total perturbation for the problem (21) is $\Gamma - S^0 R = (\Delta S) R$ and is bounded by the following quantity*

$$\mathscr{E} \leq (||\Gamma||_F + ||\Delta \Gamma||_F)\frac{||R^{-1}\Delta R||_F}{1 - ||R^{-1}\Delta R||_F} + ||\Delta \Gamma||_F \qquad (23)$$

when $||R^{-1}\Delta R||_F < 1$.

Proof.

$$\begin{aligned} \Gamma &= SR \\ \Rightarrow \Gamma &= (S^0 + \Delta S)R \\ \Rightarrow \Gamma - S^0 R &= (\Delta S)R \end{aligned}$$

$(\Delta S)R$ is called total perturbation.

We have

$$A^{-1} - (A+E)^{-1} = \sum_{k=1}^{\infty} (-1)^{k+1} (A^{-1}E)^k A^{-1} \tag{42}$$

Apply (42) to $A = R, E = \Delta R = R^0 - R$, we have

$$R^{-1} - (R + R^0 - R) = \sum_{k=1}^{\infty} (-1)^{k+1} (R^{-1}\Delta R)^k R^{-1}$$

Also,

$$\Gamma^0 = S^0 R^0 \tag{43}$$

$$\Rightarrow S^0 = \Gamma^0 (R^0)^{-1} \tag{44}$$

$$S = \Gamma R^{-1} \tag{45}$$

$$\Rightarrow \Delta S = \Gamma R^{-1} - \Gamma^0 (R^0)^{-1} \tag{46}$$

$$= (\Gamma^0 + \Delta\Gamma) R^{-1} - \Gamma^0 (R^0)^{-1} \tag{47}$$

$$= \Gamma^0 (R^{-1} - (R^0)^{-1}) + \Delta\Gamma R^{-1} \tag{48}$$

$$= (\Gamma - \Delta\Gamma)(R^{-1} - (R^0)^{-1}) + \Delta\Gamma R^{-1} \tag{49}$$

$$(\Delta S)R = ((\Gamma - \Delta\Gamma)(R^{-1} - (R^0)^{-1}) + \Delta\Gamma R^{-1})R \tag{50}$$

$$= (\Gamma - \Delta\Gamma)(R^{-1} - (R^0)^{-1})R + \Delta\Gamma \tag{51}$$

Therefore,

$$
\begin{aligned}
(\Delta S)R &= (\Gamma - \Delta\Gamma)(R^{-1} - (R^0)^{-1})R + \Delta\Gamma \\
&= (\Gamma - \Delta\Gamma)(\sum_{k=1}^{\infty}(-1)^{k+1}(R^{-1}\Delta R)^k R^{-1})R + \Delta\Gamma \text{ (if } ||R^{-1}\Delta R||_F < 1) \\
||(\Delta S)R||_F &\leq (||\Gamma||_F + ||\Delta\Gamma||_F)(\sum_{k=1}^{\infty}(||R^{-1}\Delta R||_F)^k) + ||\Delta\Gamma||_F \\
&= (||\Gamma||_F + ||\Delta\Gamma||_F)\frac{||R^{-1}\Delta R||_F}{1 - ||R^{-1}\Delta R||_F} + ||\Delta\Gamma||_F
\end{aligned}
$$

□

Similar to Theorem 4, the total perturbation ε_i for the problem (22) can be estimated using the following theorem.

Theorem 5. *ε_i can be bounded as follows:*

$$\varepsilon_i = ||((\Delta S)R)_i'|| \leq \frac{||R^{-1}\Delta R||_F}{1 - ||R^{-1}\Delta R||_F} \left\|[(\Gamma - \Delta\Gamma)']_i\right\| + \left\|(\Delta\Gamma')_i\right\| \quad (24)$$

or

$$\varepsilon_i = ||((\Delta S)R)_i'|| \approx ||R^{-1}\Delta R|| \left\|[(\Gamma - \Delta\Gamma)']_i\right\| + \left\|(\Delta\Gamma')_i\right\| \quad (25)$$

The following proposition suggests that the network structure can be estimated via an averaging procedure along the lines of Proposition 1.

Proposition 2. *Let $\hat{\Gamma}(t_r), (r = 1..N)$ be a measurement or approximation of the total influence matrix $\Gamma^0(t)$ at time t_r. Let $\hat{S}(t_r)$ be the direct influence matrix computed from $\hat{\Gamma}(t_r)$ using different methods, including ND or ℓ_1-min formulation with different bounds and $\bar{S}^{(N)} = \frac{1}{N}\sum_{r=1}^{N}\hat{S}(t_r)$. Then $\forall(i,j)$ satisfying $s_{ij}^0(t) = 0, \forall t$, $E|\bar{S}_{ij}^{(N)}|^2 \to 0$ as $N \to \infty$.*

Proof. Under perfect reconstruction per ND, $\hat{S} = S^0 + \Delta S$ satisfies

$$\hat{S}R = \Gamma$$

For the rth realization of $\Delta\Gamma_r$, ΔR_r, we have

$$
\begin{aligned}
\hat{S}(t_r)(R^0 + \Delta R_r) &= \Gamma_r^0 + \Delta\Gamma_r \\
\Rightarrow \hat{S}(t_r) &= (\Gamma_r^0 + \Delta\Gamma_r)(R^0(t_r) + \Delta R_r)^{-1} \quad (52)
\end{aligned}
$$

As $(\Delta\Gamma_{r_1}, \Delta R_{r_1})$ and $(\Delta\Gamma_{r_2}, \Delta R_{r_2})$ are independent if $r_1 \neq r_2$, $\hat{S}_{r_1}$ and $\hat{S}_{r_2}$ are independent. Therefore,

$$Var((\bar{S}^{(N)})_{ij}) = \frac{1}{N^2} Var(\sum_{r=1}^{N} (\hat{S}(t_r))_{ij}) = \frac{1}{N^2} \sum_{r=1}^{N} Var((\hat{S}(t_r))_{ij})$$

Assume that $Var((\hat{S}(t_r))_{ij})$ are bounded by a constant C, for all r,

$$\begin{aligned} Var((\bar{S}^{(N)})_{ij}) &\leq \frac{1}{N^2} NC = \frac{C}{N} \\ \Rightarrow Var((\bar{S}^{(N)})_{ij}) &\rightarrow 0 \text{ as } N \rightarrow \infty. \end{aligned}$$

□
