# Supplementary material for: Inferring sparse networks for noisy transient processes
Source: Sci Rep. 2016 Feb 26;6:21963. doi: 10.1038/srep21963 (PMC4768174; doi:10.1038/srep21963)
Supplement: Supplementary Information [file srep21963-s2.doc]

% Copy the part of code in between "Begin code to generate figure xxx" and

% "End Code to generate figure xxx" and paste on a matlab file and run it.

% Data is from DREAM5 challenge

% This code also requires l1 magic package: http://statweb.stanford.edu/~candes/l1magic/ and the Network deconvolution code.

%% -----------------Begin code to generate figure 2------------------------------

function fig2

clc

comparel1_decolvo_silencing_bcs_call1(100,0.70,0.05)

end

function G= G_from_S(S)

G=inv(-1*S+eye(size(S,1)))*S;

end

function S = S_from_G(G)

S=G*inv(G+eye(size(G,1)));

end

function H=Hoyer(A)

tem=reshape(A,[],1);

n=size(tem,1);

H=(sqrt(n)-sum(abs(tem))/sqrt(sum(tem.*tem)))/(sqrt(n)-1);

%H=(sqrt(n) - sum(abs(A)))/sqrt(sum(sum(A.*A))))/(sqrt(n)-1);

end

function comparel1_decolvo_silencing_bcs_call1(n,c,alpha)

%Study effect of number of measurements to error

nruns=10;

i=1;

while i<=nruns

muS=randn/25;

sigmaS=abs(randn)/25;

muSstored(i)=muS;

sigmaSstored(i)=sigmaS;

S=generate_scale_free(muS,sigmaS,n);%original S

G=G_from_S(S);

A=abs(G)>0;

deltaG=alpha*(muS+sigmaS*randn(n));

Ghat=G+deltaG;%noised G

if abs(norm(Ghat))> 1

continue

end

stored_Ghat(:,:,i)=Ghat;

stored_deltaG(:,:,i)=deltaG;

%S_BCS_Lap=BCS_lap2(Ghat);

Sdecolvo=S_from_G(Ghat);

tic

%[Sl11,Sl12,Sl13,Sl10,Sirls,Slasso{i}]=solvel1(Ghat,S);

[Sl11,Sl12,Sl13,Sl10,Sl123,Sl124,Sl125,sumepsilon(i),sumepsilon1(i),sumepsilon2(i)]=solvel1v1(Ghat,S,n,deltaG);

toc

Ssilencing = direct_silencing(Ghat);

Sdecolvotem=0*Sdecolvo;

deltaSl11=reshape(Sl11-S,[],1);

deltaSl12=reshape(Sl12-S,[],1);

deltaSl123=reshape(Sl123-S,[],1);

deltaSl124=reshape(Sl124-S,[],1);

deltaSl125=reshape(Sl125-S,[],1);

deltaSl13=reshape(Sl13-S,[],1);

deltaSl10=reshape(Sl10-S,[],1);

deltaSdecolvo=reshape(Sdecolvo-S,[],1);

deltaSsilencing=reshape(Ssilencing-S,[],1);

%deltaSirls=reshape(Sirls-S,[],1);

%deltaS_BCS=reshape(S_BCS_Lap-S,[],1);

Scolumn=reshape(S,[],1);

Sl11errorrate(i)=norm(deltaSl11)/norm(Scolumn);

Sl12errorrate(i)=norm(deltaSl12)/norm(Scolumn);

Sl123errorrate(i)=norm(deltaSl123)/norm(Scolumn);

Sl124errorrate(i)=norm(deltaSl124)/norm(Scolumn);

Sl125errorrate(i)=norm(deltaSl125)/norm(Scolumn);

Sl13errorrate(i)=norm(deltaSl13)/norm(Scolumn);

Sl10errorrate(i)=norm(deltaSl10)/norm(Scolumn);

%Sirlserrorrate(i)=norm(deltaSirls)/norm(Scolumn);

Sdecolvoerrorrate(i)=norm(deltaSdecolvo)/norm(Scolumn);

Ssilencingerrorrate(i)=norm(deltaSsilencing)/norm(Scolumn);

en_decolvo(i)=entropy(Sdecolvo)

en_Sl11(i)=entropy(Sl11)

en_Sl12(i)=entropy(Sl12)

en_Sl123(i)=entropy(Sl123)

en_Sl124(i)=entropy(Sl124)

en_Sl125(i)=entropy(Sl125)

en_Sl10(i)=entropy(Sl10)

Hoyer_decolvo(i)=Hoyer(Sdecolvo)

Hoyer_Sl11(i)=Hoyer(Sl11)

Hoyer_Sl12(i)=Hoyer(Sl12)

Hoyer_Sl123(i)=Hoyer(Sl123)

Hoyer_Sl124(i)=Hoyer(Sl124)

Hoyer_Sl125(i)=Hoyer(Sl125)

Hoyer_Sl10(i)=Hoyer(Sl10)

i=i+1;

end

figure;hist([Sl124errorrate',Sl125errorrate',Sdecolvoerrorrate']);

t={'$l_1\min$ using $\varepsilon_0$','$l_1\min$ using $\varepsilon_1$','Decolvolution method'}

legend(t,'interpreter', 'latex')

axis tight;

figure;hist([Hoyer_Sl124',Hoyer_Sl125',Hoyer_decolvo']);

t={'$l_1\min$ using $\varepsilon_0$','$l_1\min$ using $\varepsilon_1$','Decolvolution method'}

legend(t,'interpreter', 'latex')

axis tight;

end

function H=entropy(A)

B=abs(A/norm(A,2));

H=-sum(B(B~=0).*log(B(B~=0)));

end

function [Sl11,Sl12,Sl13,Sl10,Sl123,Sl124,Sl125,sumepsilon,sumepsilon1,sumepsilon2]=solvel1v1(Ghat,S,nmeasures,deltaG)%This solve the deconvolution using l1 formulation

%check formula for total perturbation and how it connects with the l1 min

%formulation. Does it help in defining epsilon in the l1 min formulation

%problem?

Ghatt=Ghat';

n1=size(Ghatt,1);

A1=(eye(n1)+Ghatt);

nmeasures=n1;n=n1;

A=A1(1:nmeasures,:);

sumepsilon=0;

sumepsilon1=0;

sumepsilon2=0;

for i=1:n1

%tic

y=Ghatt(1:nmeasures,i)

x0 = A'*y;

x00=S(i,:);

epsilon=norm(A*x00'-y);

stored_epsilon(i)=epsilon;

%epsilon1=(1/sqrt(n))*norm(Ghat+eye(n),'fro')*norm(deltaG,'fro')/((1-norm(Ghat,'fro')-norm(deltaG,'fro'))*(1-norm(Ghat,'fro')))

epsilon1=(1/sqrt(n))*(norm(Ghat,'fro')+1)*norm(deltaG,'fro')/((1-norm(Ghat,'fro')-norm(deltaG,'fro'))*(1-norm(Ghat,'fro')))

epsilon2=(norm(Ghat,'fro')+1)*(1/sqrt(n))*max(abs(eig(deltaG)))

epsilon3=(norm(Ghat,'fro')+1)*(1/sqrt(n))*(norm(deltaG,2)+norm(deltaG,2)^2)

stored_epsilon3(i)=epsilon3;

stored_epsilon2(i)=epsilon2;

stored_epsilon1(i)=epsilon1;

epsilon3=(norm(Ghat,'fro')+1)*(1/sqrt(n))*norm(deltaG,2);

x1=l1qc_logbarrier(x0,A,[],y,epsilon1);

x2=l1qc_logbarrier(x0,A,[],y,2*epsilon1);

x23=l1qc_logbarrier(x0,A,[],y,1/2*epsilon1);

x24=l1qc_logbarrier(x0,A,[],y,epsilon);

x25=l1qc_logbarrier(x0,A,[],y,epsilon3);

x3=l1qc_logbarrier(x0,A,[],y,epsilon3/2);

x01=l1qc_logbarrier(x0,A,[],y,epsilon3/10);

xirls=0;%irls1d(A,y,1);

S1t(:,i)=x1;

S2t(:,i)=x2;

S23t(:,i)=x23;

S24t(:,i)=x24;

S25t(:,i)=x25;

S3t(:,i)=x3;

S0t(:,i)=x01;

end

Sl11=S1t';

Sl12=S2t';

Sl13=S3t';

Sl123=S23t';

Sl124=S24t';

Sl125=S25t';

Sl10=S0t';

sumepsilon=sum(stored_epsilon.^2);

sumepsilon1=sum(stored_epsilon1.^2);

sumepsilon2=sum(stored_epsilon2.^2);

%Sirls=Sirlst';

end

function S=direct_silencing(G)

n=size(G,1);

S=(G-eye(n)+diag(diag((G-eye(n))*G)))*pinv(G);

end

function S=random_DAG_generate(muS,sigmaS,n,c)

dag = randperm(n);

S=zeros(n);

for i=1:n

for j=i+1:n

%S(dag(i),dag(j))=1.5*(rand-0.5)*double(rand>c);

S(dag(i),dag(j))=(muS+sigmaS*randn)*double(rand>c);

%S(dag(i),dag(j))=0.07*double(rand>c);

end

end

end

function x=IHT(xn,Phi,y,lambda)

tem=xn + Phi'*(y-Phi*xn);

x=(abs(tem)>sqrt(lambda)).*tem;

end

function plotSG()

h=figure;

subplot(2,1,1);

hist(reshape(S,1,[]),100)

%set(gca, 'YScale', 'log')

title('Histogram of S');

subplot(2,1,2)

hist(reshape(G,1,[]),100)

%set(gca, 'YScale', 'log')

title('Histogram of G');

%Hoang.output_standardize_figure(h,['2_distribution_alpha',num2str(alpha)],'.eps');

figure;

%Hoang.output_standardize_figure(h3,['2_compare_l1_deconvo_alpha',num2str(alpha)],'.eps');

end

function S=generate_scale_free(muS,sigmaS,n)

%% Genrate the network:

NumberOfNodes = n; % Number of nodes

Alpha = -2.2; % Alpha of the scale-free graph

%define node degree distribution: From the paper: http://www.nature.com/nature/journal/v407/n6804/pdf/407651a0.pdf

% genetic regulatory network is scale free http://www.sciencemag.org/content/303/5659/808.full

XAxis = unique(round(logspace(0,log10(NumberOfNodes),25)));

YAxis = unique(round(logspace(0,log10(NumberOfNodes),25))).^(Alpha+1);

% create the graph with the required node degree distribution:

Graph = mexGraphCreateRandomGraph(NumberOfNodes,XAxis,YAxis,1);

S=zeros(NumberOfNodes);

for i=1:length(Graph.Data)

S(Graph.Data(i,1),Graph.Data(i,2))=(muS+sigmaS*randn);

end

end

function plotsfn(Graph)

%% Node Degree Distribution:

Degrees = GraphCountNodesDegree(Graph);

h1 = figure;

% incoming:

[y x] = hist(Degrees(:,2),unique(Degrees(:,2)));

loglog(x,y/sum(y),'*r');

hold on

% outgoing

[y x] = hist(Degrees(:,3),unique(Degrees(:,3)));

loglog(x,y/sum(y),'dg');

% expected distribution:

%loglog(XAxis,YAxis/sum(YAxis),':b');

xlabel('k,Degree');

ylabel('P(k)');

title('Node Degree Distribution');

legend({'Incoming','Outgoing'});

end

%% -------------------End Code to generate figure 2-------------

%% -------------------Begin Code to generate figure 3-------------

function fig3

mean_error_vaepsilon_rate(100,0.90,0.5)

end

function S=generate_scale_free(muS,sigmaS,n)

%% Genrate the network:

NumberOfNodes = n; % Number of nodes

Alpha = -2.2; % Alpha of the scale-free graph

%define node degree distribution: From the paper: http://www.nature.com/nature/journal/v407/n6804/pdf/407651a0.pdf

% genetic regulatory network is scale free http://www.sciencemag.org/content/303/5659/808.full

XAxis = unique(round(logspace(0,log10(NumberOfNodes),25)));

YAxis = unique(round(logspace(0,log10(NumberOfNodes),25))).^(Alpha+1);

% create the graph with the required node degree distribution:

Graph = mexGraphCreateRandomGraph(NumberOfNodes,XAxis,YAxis,1);

S=zeros(NumberOfNodes);

for i=1:length(Graph.Data)

S(Graph.Data(i,1),Graph.Data(i,2))=(muS+sigmaS*randn);

end

end

function mutual_coh=mutual_coh(B)

n=size(B,1);

mutual_coh=0;

for i=1:n-1

for j=i+1:n

mutual_coh=max(abs(mutual_coh),B(:,i)'*B(:,j)/(norm(B(:,i))*norm(B(:,j))));

end

end

end

function G= G_from_S(S)

G=inv(-1*S+eye(size(S,1)))*S;

end

function S = S_from_G(G)

S=G*inv(G+eye(size(G,1)));

end

function mean_error_vaepsilon_rate(n,c,alpha)

%Study effect of (varepsilon/varepsilon_0) to mean error

rates=[0.01 0.1 0.5 1 2 3 4 5];

nruns=40;

i=1;

while i<=nruns

muS=randn/25;

sigmaS=abs(randn)/25;

S=generate_scale_free(muS,sigmaS,n);%original S

G=G_from_S(S);

deltaG=alpha*(muS+sigmaS^2*randn(n));

Ghat=G+deltaG;%noised G

if abs(norm(Ghat,'fro'))> c

continue

end

if abs(norm(Ghat,'fro')+norm(deltaG,'fro'))> c

continue

end

for irate=1:length(rates)

rate=rates(irate);

error_rate(irate,i)=solvel1v1(Ghat,S,rates(irate));

end

i=i+1;

end

mean_error=mean(error_rate,2);

save('mean_error_test.mat','mean_error');

figure;

plot(rates,mean_error,rates,mean_error,'b*')

t='$\frac{\varepsilon}{\varepsilon_0}$';

xlabel(t,'interpreter', 'latex')

t='mean error';

ylabel(t,'interpreter', 'latex')

%Hoang.save_standized_fig(gcf,'fig3_test')

end

function H=entropy(A)

B=abs(A/norm(A,2));

H=-sum(B(B~=0).*log(B(B~=0)));

end

function [error_rate]=solvel1v1(Ghat,S,rate)%

Ghatt=Ghat';

n1=size(Ghatt,1);

A=(eye(n1)+Ghatt);

for i=1:n1

y=Ghatt(:,i)

x0 = A'*y;

x00=S(i,:);

epsilon0=norm(A*x00'-y);

xl1=l1qc_logbarrier(x0,A,[],y,rate*epsilon0);

Sl1_0t(:,i)=xl1;

end

Sl1_0=Sl1_0t';

error_rate=norm(reshape(Sl1_0-S,[],1))/norm(reshape(S,[],1));

end

function S=direct_silencing(G)

n=size(G,1);

S=(G-eye(n)+diag(diag((G-eye(n))*G)))*pinv(G);

end

function H=Hoyer(A)

tem=reshape(A,[],1);

n=size(tem,1);

H=(sqrt(n)-sum(abs(tem))/sqrt(sum(tem.*tem)))/(sqrt(n)-1);

end

%% -------------------Eegin Code to generate figure 3-----------

%% -------------------Begin Code to generate figure 5-----------

% New script to validate my work using real data sets and simulation

function fig5

dbstop if error;

clc;

%test()

for ij=1:2

ValidateUsingODES(ij);

end

for ij=4:5

ValidateUsingODES(ij);

end

%numerictype(1, 16, 15)

end

%1. Simulation using ODEs

function ValidateUsingODES(ij)

% 1. Generate initial condition and network structure

tic

nSim=30;

for iSim=1:nSim

tic;

id=randperm(85,85);

id=id(1:40);

id=sort(id);

id=id(10:30);

LoadOrSim=1;

if LoadOrSim==1

N=40; %number of nodes

L = 10; %number of edges/ links

x0=10*abs(randn(N,1));

muS0=5; sigmaS0=0.50;

S0=generate_scale_free(muS0,sigmaS0,N+2);

T=[0:0.01:1];

tol=1e-9*ones(size(S0,1),1);

options = odeset('RelTol',1e-9,'AbsTol',tol);

deltap=0.5;

% save('..\DataFigures\odes_testing7.mat');

%save('..\DataFigures\odes_goodsetting.mat');

else

load('..\DataFigures\odes_testing5.mat');

end

deltap=0.04;%0.4 is really good.

T=[0:0.01:6];alpha0=-5*abs(randn(1,N));

alpha=alpha0.*ones(1,N);

[t,x]=ode45(@(t,x)xdot(alpha,S0,x),T,x0,options);

sigma=10^(-1*ij);

noise1=sigma*randn(size(x));

x=x+noise1;

it0=1;it1=400

for i=1:N %To compute coeficients of the iRow row.

sprintf('i=%d,iSim=%d',i,iSim)

%compute perturbation set.

idset=(1:N);

if i==1,idset2=idset+1;idset2(N)=1;idset(i)=N;end

if i==N,idset(i)=N-1;idset2=idset+1;idset2(N)=1;end

if ((1<i)&(i<N)), idset(i)=1;idset2=idset+1;idset2(N)=1;idset2(i)=N;end

for j=1:N %j in Pi/idset

S1=zeros(N);

S1(idset(j),idset2(j))=deltap;

S1=S1+S0;

if exist('xperturb','var'), clear('xperturb');end

[t,xperturb] = ode45(@(t,xperturb)xdot(alpha,S1,xperturb),T,x0,options);

noise2=sigma*randn(size(xperturb));

xperturb=xperturb+noise2;

for k=1:N

for it=it0:length(t)

R(k,j,it)=(xperturb(it,k)-x(it,k))/deltap;%formula (6)

noiseR(k,j,it)=(noise2(it,k)-noise1(it,k))/deltap;

end

end

end

%add one more line for xi

S1=zeros(N);

if exist('xperturb','var'), clear('xperturb');end

alpha=alpha0.*ones(1,N);

alpha(i)=alpha0(i)+deltap;

[t,xperturb] = ode45(@(t,xperturb)xdot(alpha,S0,xperturb),T,x0,options);

noise2=sigma*randn(size(xperturb));

xperturb=xperturb+noise2;

for k=1:N

for it=it0:length(t)

Re(k,it)=(xperturb(it,k)-x(it,k))/deltap;%formula (6)

noiseRe(k,it)=(noise2(it,k)-noise1(it,k))/deltap;

end

end

% compute rho and R

for it=it0:it1

rho(:,it)=(R(i,:,it+1)-R(i,:,it))/(t(it+1)-t(it));

noiseRho(:,it)=(noiseR(i,:,it+1)-noiseR(i,:,it))/(t(it+1)-t(it));

end

for it=it0:it1

rho(:,it)=(R(i,:,it+1)-R(i,:,it))/(t(it+1)-t(it));

noiseRho(:,it)=(noiseR(i,:,it+1)-noiseR(i,:,it))/(t(it+1)-t(it));

rho_e(it)=(Re(i,it+1)-Re(i,it))/(t(it+1)-t(it))-x(it,i)';

noise_rho_e(it)=(noiseRe(i,it+1)-noiseRe(i,it))/(t(it+1)-t(it));

end

% compute Fik, both using the method in the paper and theoretical value.

for i_id=1:length(id);

it=id(i_id);

tem=pinv(400*R(:,:,it)')*(400*rho(:,it));

A=R(:,:,it)';y=rho(:,it)';noisey=noiseRho(:,it)';

ST = dbstack;

if i<N,

Rnew=[Re(:,it),R(:,2:end,it)]';

noiseRnew=[noiseRe(:,it),noiseR(:,2:end,it)]';

else

Rnew=[Re(:,it),R(:,1:end-1,it)]';

noiseRnew=[noiseRe(:,it),noiseR(:,1:end-1,it)]';

end

ye=[rho_e(it),y(2:end)];

noiseye=[noise_rho_e(it),noisey(2:end)];

RdeltaR=inv(Rnew)*noiseRnew;%transpose or not?

RdeltaR=sqrt(max(abs(eig(RdeltaR'*RdeltaR))));

bound1 = (norm(ye)+norm(noiseye))*RdeltaR/(1-RdeltaR)+norm(noiseye);

RdeltaR1=inv(Rnew)*noiseRnew;%

RdeltaR1=sqrt(max(abs(eig(RdeltaR1'*RdeltaR1))));

bound2 = (norm(ye)+norm(noiseye))*RdeltaR1+norm(noiseye);%new approximation

RdeltaR3=0.5*inv(Rnew)*noiseRnew;

RdeltaR3=sqrt(max(abs(eig(RdeltaR3'*RdeltaR3))));

bound3 = (norm(ye)+norm(0.5*noiseye))*RdeltaR3/(1-RdeltaR3)+norm(0.5*noiseye);%should be equal to the theory value below

RdeltaR4=2*inv(Rnew)*noiseRnew;

RdeltaR4=sqrt(max(abs(eig(RdeltaR4'*RdeltaR4))));

bound4 = (norm(ye)+norm(2*noiseye))*RdeltaR4/(1-RdeltaR4)+norm(2*noiseye);%should be equal to the theory value below

%compute theoretical bound:

tem1(i,:,i_id)=pinv(Rnew)*ye';

tem1_old(i,:,i_id)=pinv(R(:,:,it))*y';

a=S0(i,:);

theory=a.*(1./(1+x(it,:)).^2);

theory(i)=theory(i)+alpha0(i);

tol=10^-8;

epsilontheory=norm(Rnew*theory'-ye',2);

epsilontheory1=norm(Rnew*theory'-ye',2);

tem_l1qc_logbarriere0(i,:,i_id)=l1qc_logbarrier(x0, Rnew, [], ye', epsilontheory1);%originally is bound 0, is the right bound

tem_l1qc_logbarriere1(i,:,i_id)=l1qc_logbarrier(x0, Rnew, [], ye', 10*epsilontheory1);

tem_l1qc_logbarriere2(i,:,i_id)=l1qc_logbarrier(x0, Rnew, [], ye', 0.1*epsilontheory1);

%tem_l1qc_logbarriere4(i,:,i_id)=l1qc_logbarrier(x0, Rnew, [], ye', bound4);

boundall3(iSim,i,i_id)=bound3;%originally is bound 0, is the right bound

boundall1(iSim,i,i_id)=bound1;

boundall2(iSim,i,i_id)=bound2;

boundall4(iSim,i,i_id)=bound4;

boundall0(iSim,i,i_id)=norm(Rnew*theory'-ye');

% compare the 4 ways of estimating

%[tem_l1qc_logbarriere0(i,:,i_id)',tem_l1qc_logbarriere1(i,:,i_id)',tem_l1qc_logbarriere2(i,:,i_id)',theory',tem1(i,:,i_id)']

end

end

%save('tem1.mat','tem1','S0');

[norm_inv,norm_inv_mean]=compare_norm(tem1,S0);

[norm_inv_old,norm_inv_meanold]=compare_norm(tem1_old,S0);

[norm_theory_bound,norm_mean_theory_bound]=compare_norm(tem_l1qc_logbarriere0,S0);

[norm_overestimated_by_10,norm_mean_overestimated_by_10]=compare_norm(tem_l1qc_logbarriere1,S0);

[norm_underestimated_by_10,norm_mean_underestimated_by_10]=compare_norm(tem_l1qc_logbarriere2,S0);

%[norm4,normmean4]=compare_norm(tem_l1qc_logbarriere4,S0);

%figure;hold on;plot(norm1);plot(normmean1,'r')

error_inv(iSim)=mean(norm_inv);

error_inv_mean(iSim)=norm_inv_mean(1);

error_inv_old(iSim)=mean(norm_inv_old);

error_inv_meanold(iSim)=norm_inv_meanold(1);

error_theory_bound(iSim)=mean(norm_theory_bound);

error_mean_theory_bound(iSim)=norm_mean_theory_bound(1);

error_overestimated_by_10(iSim)=mean(norm_overestimated_by_10);

error_mean_overestimated_by_10(iSim)=norm_mean_overestimated_by_10(1);

error_underestimated_by_10(iSim)=mean(norm_underestimated_by_10);

error_mean_underestimated_by_10(iSim)=norm_mean_underestimated_by_10(1);

% errorMeanSnorm4(iSim)=mean(norm4);

% errorMeanS4(iSim)=normmean4(1);

toc

end

%[normtem_l1qc_logbarriere,normmeantem_l1qc_logbarriere]=compare_norm(tem_l1qc_logbarriere,S0);

toc

run_by='run by nw validation 10_rerun2';

save(['MeansS-Snorm1_set_of_runDec23_2015ij',num2str(ij),'.mat'])

%% Plot the figure.

load('MeansS-Snorm1_set_of_runDec23_2015ij.mat');

figure; %hold on;boxplot([errorMeanSnorm0',errorMeanSnorm1',errorMeanSnorm2'])

var{1,1}=error_theory_bound;var{1,2}=error_mean_theory_bound;

var{2,1}=error_underestimated_by_10;var{2,2}=error_mean_underestimated_by_10;

var{3,1}=error_overestimated_by_10;var{3,2}=error_mean_overestimated_by_10;

var{4,1}=norm_inv;var{4,2}=norm_inv_mean;

var{5,1}=error_inv_old;var{5,2}=error_inv_meanold;

for i=1:5

[h(i),p(i)]=ttest(var{i,2},var{i,1},'Tail','right');

sprintf('\\rho=%4.2d,\\bar{\\rho}=%4.2d',mean(var{i,1}),mean(var{i,2}))

end

i=6;[h(i),p(i)]=ttest(norm_inv,norm_inv_old,'Tail','right')

figure;

%%

lbwh = get(gcf, 'position');

figw = lbwh(3);

figh = lbwh(4);

h=subplot(2,2,1);

% ax=get(h,'Position');

% ax(4)=ax(4)+0.1;

% set(h,'Position',ax);

boxplot([error_inv_old',error_inv_meanold'],'labels',{'Without Averaging','With Averaging'})

sprintf('old mean \\rho=%2.2d,\\bar{\\rho}=%2.2d',mean(error_inv_old),mean(error_inv_meanold))

%title('Sontag''s method')

title('(a)')

%axis square

h=subplot(2,2,2);

% ax=get(h,'Position');

% ax(4)=ax(4)+0.1;

% set(h,'Position',ax);

boxplot([error_theory_bound',error_mean_theory_bound'],'labels',{'Without Averaging ',' With Averaging'})

%title('l_1-min with theoretical bound')

sprintf('theory \\rho=%2.2d,\\bar{\\rho}=%2.2d',mean(error_theory_bound),mean(error_mean_theory_bound))

title('(b)')

%axis square

h=subplot(2,2,3);

% ax=get(h,'Position');

% ax(4)=ax(4)+0.1;

% set(h,'Position',ax);

boxplot([error_underestimated_by_10',error_mean_underestimated_by_10'],'labels',{'Without Averaging','With Averaging'})

%title('l_1 min with underestimated bound')

sprintf('underestimated \\rho=%2.2d,\\bar{\\rho}=%2.2d',mean(error_underestimated_by_10),mean(error_mean_underestimated_by_10))

%title('l_1 min with overestimated bound')

title('(c)')

sprintf('overestimated \\rho=%2.2d,\\bar{\\rho}=%2.2d',mean(error_overestimated_by_10),mean(error_mean_overestimated_by_10))

%axis square

h=subplot(2,2,4);

boxplot([error_overestimated_by_10',error_mean_overestimated_by_10'],'labels',{'Without Averaging','With Averaging'})

% ax=get(h,'Position');

% ax(4)=ax(4)+0.1;

% set(h,'Position',ax);

%axis square

tightfig;

%run 3: 10^-5

% run 4: 10^-4;

end

function [norm1,normmean1]=compare_norm(tem0,S0)

%id=1:size(tem0,3);

tem1=tem0;

for item=1:size(tem1,3)

norm1(item)=norm(tem1(:,:,item).*(1-abs(S0)>0));

normmean1(item)=norm(mean(tem1,3).*(1-abs(S0)>0));

end

end

function Sk=PartialXk(S0,k,T,deltap,options,x,t)

N=size(x,1);

idset=[1:N];

for i=1:N

if k==1,idset2=idset+1;idset2(n)=1;idset(k)=n;end

if k==N,idset(k)=n-1;idset2=idset+1;idset2(N)=1;end

if ((1<k)&(k<N)), idset(k)=1;idset2=idset+1;idset2(n)=1;idset2(k)=N;end

S(idset(i),idset2(i))=S(idset(i),idset2(i))+deltap;

[ti,xperturb]=ode45(@(ti,xperturb)xdot(S0,xperturb),T,x(:,1),options);

%noise2=sigma*randn(size(x)).*xperturb;

%xperturb=xperturb+noise2;

for j=1:n

for it=length(t)-1:length(t)

R(i,j,it)=(xperturb(it,j)-x(it,j))/deltap;%formula (6)

end

end

end

end

function xdot=xdot(alpha,A,x)

xdot= alpha'.*x + A*(x./(1+x));

%xdot=A*x;

end

% Utilities functions

function S=generate_scale_free(muS,sigmaS,n)

%% Genrate the network:

%rng;

%n=n+2;

run=1;

while run==1

run=0;

NumberOfNodes = n; % Number of nodes

Alpha = -2.2; % Alpha of the scale-free graph, original 2.2

%define node degree distribution: From the paper: http://www.nature.com/nature/journal/v407/n6804/pdf/407651a0.pdf

% genetic regulatory network is scale free http://www.sciencemag.org/content/303/5659/808.full

XAxis = unique(round(logspace(0,log10(NumberOfNodes),25)));

YAxis = unique(round(logspace(0,log10(NumberOfNodes),25))).^(Alpha+1);

% create the graph with the required node degree distribution:

Graph = mexGraphCreateRandomGraph(NumberOfNodes,XAxis,YAxis,1);

S=zeros(NumberOfNodes);

for i=1:length(Graph.Data)

S(Graph.Data(i,1),Graph.Data(i,2))=(muS+sigmaS*randn);

end

%S = S(1:end-2,1:end-2);

for i=1:n-2

if max(abs(S(i,:)))==0, run=1;break;

end

% if max(abs(S(:,i)))==0, run=1;break;

% end

end

S = S(1:end-2,1:end-2);

end

end

function plotSG(S)

h=figure;

subplot(2,1,1);

hist(reshape(S,1,[]),100)

%set(gca, 'YScale', 'log')

title('Histogram of S');

subplot(2,1,2)

hist(reshape(G,1,[]),100)

%set(gca, 'YScale', 'log')

title('Histogram of G');

figure;

end

%% -------------------End Code to generate figure 5-----------

%% ------------------Begin code to generate data for figure 6-----------

function fig6_data(alpha)

Spiece={'in_silico','','Ecoli','cerevisiae'}

%Nw={'Pearson','Spearman','aracne','mi'};

Nw={'aracne','mi'};

%parpool(4);

for iSpiece=[1,3,4]

for iNw=[1,2]

b=import_dat(iSpiece,Spiece{iSpiece});

ntf=ntf_compute(iSpiece);

G=import_G(iSpiece,Nw{iNw});

n=max(size(G));

[S,scale]=ND_regulatory(abs(G));

Gob=G/scale;

Gob=abs(Gob);

Gob=Gob-Gob.*eye(size(Gob,1));

A=(eye(size(Gob,1))+Gob);

parfor itf=1:ntf,

y=Gob(:,itf);

x0 = A'*y;

a1=l1qc_logbarrier(x0,A,[],y,alpha*norm(y));

Sl1_1(:,itf)=a1;

end

save([Spiece{iSpiece},'_',Nw{iNw},'_',num2str(alpha),'.mat'],'Sl1_1');

clear('Sl1_1');

end

end

end

function G=import_G(i,nwi)

Spiece={'in_silico','','Ecoli','cerevisiae'};

G=load([Spiece{i},'_',nwi,'_net']);

if length(nwi)==2,G=G.mi_net;else

G=G.aracne_net;end

end

function b=import_dat(i,nwi)

load(['.../DREAM5_network_inference_challenge/Network',num2str(i),'/input data/',nwi]);

end

function ntf=ntf_compute(i)

filename=['.../DREAM5_network_inference_challenge/Network',num2str(i),'/input data/net',num2str(i),'_transcription_factors.tsv'];

delimiter = '';

formatSpec = '%s%[^\n\r]';

fileID = fopen(filename,'r');

dataArray = textscan(fileID, formatSpec, 'Delimiter', delimiter, 'ReturnOnError', false);

fclose(fileID);

ntf=length(dataArray{1});

end

%-------------------End code to generate data for figure 6-----------
